# The Dark Side of the Hospitality Industry: Workplace Bullying and Employee Well-Being with Feedback Avoidance as a Mediator and Psychological Safety as a Moderator

**DOI:** 10.3390/healthcare13030319

**Published:** 2025-02-04

**Authors:** Ibrahim A. Elshaer, Alaa M. S. Azazz, Mohammed E. A. Zain, Sameh Fayyad, Noha Ismaeil ElShaaer, Samy Wageh Mahmoud

**Affiliations:** 1 Department of Management, College of Business Administration, King Faisal University, Al-Ahsaa 380, Saudi Arabia; 2 Department of Social Studies, Arts College, King Faisal University, Al-Ahsaa 380, Saudi Arabia; 3 Public Law Department, Faculty of Law, King Faisal University, Al-Ahsaa 380, Saudi Arabia; melsadig@kfu.edu.sa; 4 Hotel Studies Department, Faculty of Tourism and Hotels, Suez Canal University, Ismailia 41522, Egypt; sameh.fayyad@tourism.suez.edu.eg (S.F.); samy_wageh@tourism.suez.edu.eg (S.W.M.); 5 Hotel Management Department, Faculty of Tourism and Hotels, October 6 University, Giza 12573, Egypt; noha.saadel-di.tou@o6u.edu.eg

**Keywords:** workplace bullying, hospitality industry, hotel industry, feedback avoidance, psychological safety, well-being

## Abstract

Objectives: The tourism and hospitality industry, well-known as a people-oriented industry, is not immune to the adverse outcomes of workplace bullying. This paper explores the darker side of the tourism and hospitality sector by investigating workplace bullying and its potential impact on shaping employee well-being. Specifically, the study explores how feedback avoidance can mediate the relationship between information flow and employee well-being and how psychological safety can moderate the relationship between information flow and employee well-being in bullying contexts. Methods: Using a quantitative-methods approach, the paper analyzed survey data from 341 employees at five-star hotels in Sharm El-Sheikh, Egypt, with structural equation modelling (PLS-SEM program). Results: The findings indicated that the spread of information about workplace bullying promotes the feelings of stress among employees which negatively affects their wellbeing in the workplace. Additionally, feedback avoidance as a mediator was found to foster the harmful impacts of bullying. Conversely, psychological safety as a moderator functioned as a protective element, mitigating the negative influence of workplace bullying on employees’ well-being. Conclusions: This paper enhanced our understanding of the dark side of the hospitality industry, specifically workplace bullying, by highlighting the key role of information dynamics about bullying in the workplace and the role of psychological safety in shaping overall employee well-being.

## 1. Introduction

The prevalence of information about bullying dramatically differs from industry to industry [1,2]. The intricate work environment in the hospitality industry, which includes volatile employment, uncomfortable working environments, stressful service situations, poor wages, long and unsocial work hours, emotional labor, the failure to adopt formal HRM mechanisms, and power imbalance, makes it a fertile environment for high rates of bullying incidents, unlike other businesses [3,4]. A growing number of studies confirmed that work bullying, which implies repeated aggressive information and hostile acts directed at employees [5,6], was positively related to emotional exhaustion [7], psychological stress [8], burnout and higher intentions to quit [9], decrease staff positive coping capabilities [4], as well as psychological/mental health problems such as depression and anxiety [10,11] and even suicide [12] and was negatively associated with employees’ wellbeing [13,14].

Employee well-being is a term that “everyone understands the meaning of, but nobody can precisely define” [15]. Generally, it is associated with happiness (i.e., hedonism), the fulfillment of human potential (i.e., eudaimonism), and emotional, psychological, and social well-being [16]. Researchers have worked to investigate factors that enhance or hinder employees’ well-being because positive feelings make them happy and more productive at work [17]. In this context, many previous studies analyzed the devastating consequences that information about workplace bullying can have on employees’ well-being. Research has demonstrated that exposure to bullying at work is linked to poorer well-being, with considerable effect sizes ranging from medium to significant [18,19,20]. As per the Job Demands–Resources (JD-R) model, workplace bullying can be seen as a job demand since it requires significant cognitive and emotional efforts from the targets to deal with the condition, explaining why it negatively affects employees’ well-being [21]. Bullying at work has a detrimental and damaging effect on employees’ well-being, which lowers productivity and service quality. Employers must, therefore, endeavor to eradicate bullying in the workplace. However, the literature studying the consequences of workplace bullying on employee well-being requires more attention to be paid to investigating the nature of the relationship, particularly in the hospitality field [13,22].

Meanwhile, scholars have recently begun analyzing employees’ passive coping techniques (feedback avoidance) in reaction to workplace mistreatment (workplace bullying) [23,24]. According to Qian et al. (2017), avoidance behavior is a coping mechanism used to lessen the discomfort a potentially threatening person or circumstance brings [25]. Moreover, the negative feelings resulting from workplace bullying may impel targets to participate in behaviors planned to avoid further bullying encounters. Consequently, feedback avoidance can act as a means for bullied employees to cope with these conditions to defend their limited resources by escaping further bullying situations, which, in turn, decreases employee well-being. Accordingly, the study strived to explore the mediating role of employee feedback avoidance in the relationship between bullying in the workplace and employee well-being. Although investigators and practitioners are constantly interested in avoiding feedback behavior [26], to the authors’ knowledge, studies have largely neglected to investigate this relationship which contributes to this study.

To mitigate the impact of bullying in the workplace, scholars have called for a greater focus on variables that can moderate the effect of workplace bullying [18,19,27]. The present study used psychological safety as a moderator in the proposed model, arguing that employees in a psychologically safe workplace have good intentions toward each other, can have constructive conflicts, entrust one another to not discard them for expressing their opinions, and feel comfortable taking chances and trying new things. Furthermore, psychological safety enables individuals to emphasize group goals and problem avoidance more than self-defense [28]. Explicitly, studies, depending on the JD-R model, proved that psychological safety in the workplace assisted in preventing bullying [28,29]. Yet, little investigation has explored the psychological safety variable as a moderator in workplace bullying-affected paths despite it being a crucial determinant of health issues and employee safety matters.

Generally, investigations on analyzing mediated/moderated links between workplace bullying and its possible outcomes are necessary [27,30]. There is a continuing call to explore not only the antecedents but also the consequences of workplace bullying across cultures and working conditions, especially in the hospitality industry and, specifically, in developing countries [3,30].

According to the JD-R model, job characteristics encompass job demands, which require sustained physical and mental effort, and job resources, which include physical, psychological, and organizational factors that support goal attainment [31]. The effects of workplace bullying, including workload, job insecurity, role conflict, poor communication, and low social support, represent job demands. In contrast, the effects of psychological safety, such as task autonomy, social support, and high job control, represent job resources. Therefore, to address the study gaps, the study, by applying the JD-R model, aimed to achieve the following five research objectives: (1) examine the direct relationships among workplace bullying (WB), employee well-being behavior (EWB), and employee feedback avoidance behavior (FAB); (2) examine the mediating role of FAB in the relationship between WB and EWB; (3) determine whether psychological safety (PS) moderates the effect of WB on FAB; (4) assess whether PS moderates the effect of FAB on EWB; and (5) in light of the findings, the study aims to provide a set of practical theoretical applications targeted at hotel managers in developing countries.

## 2. Theoretical Base and Hypotheses Justification

### 2.1. Workplace Bullying (WB) and Employee Well-Being (EWB)

The hotel business is characterized by its labor-intensive nature, job-demanding, and hierarchical structure. As a result, WB targeting vulnerable employees, including trainees, frontline staff, and entry-level workers, is often considered commonplace or even an accepted aspect of the job [32,33]. Previous studies have indicated that workplace bullying behaviors in the hospitality business, often involving excessive workloads, workplace incivility, ignored opinions, and sexual harassment [34], are a major contributor to employee stress and psychological harm [23]. Generally, bullying in the workplace refers to “situations where a person repeatedly, and over a period of time, is exposed to negative acts on the part of coworkers, supervisors, or subordinates” [35]. The negative impacts of WB often escalate because it is commonly characterized by a repeated intention to damage the social life, reputation, or performance, of the targeted individual [14]. Therefore, by operating the conservation of resources (COR) theory [36], WB may be a higher risk to employee well-being due to the stress caused by the loss of resources for bullied employees [13]. Here, previous studies have called for examining the effects of WB, especially on employee well-being, in non-Western contexts, such as developing countries [37]. Analyzing the effects of abusive workplace behaviors, including WB, on employee well-being is crucial, as well-being is positively associated with organizational loyalty and commitment [38]. Thus, hypothesis 1 can be written as below.

**Hypothesis** **1 (H1).**  *Workplace bullying can negatively influence employee well-being.*

### 2.2. Workplace Bullying (WB) and Feedback Avoidance Behavior (FAB)

Feedback avoidance behavior is defined as “the extent to which employees use strategies that are designed to either totally avoid their supervisors or divert their supervisor’s attention so that their poor performance is not acknowledged, and they do not receive negative verbal feedback” [39]. According to [40], also, feedback avoidance is a deliberate, positive, and determined feedback management strategy that implies “active behaviours directed at evading feedback”. Feedback plays a vital role in building and sustaining a higher performance workplace by anticipating and addressing potential issues [41]. Conversely, feedback avoidance behavior adversely impacts followers’ performance and well-being [40].

Previous research confirmed that destructive leadership, including bullying as one of its tactics, is a key predictor of feedback avoidance behavior [42]. Based on the COR theory [36], bullied employees might be either unable or hesitant to respond to supervisory bullying with aggressive retaliatory behaviors, given their reliance on the supervisor for critical resources like promotions and job security. Instead, avoidance may function as a less confrontational coping strategy and a way to preserve their resources [43]. Thus, feedback avoidance behavior, as a passive coping strategy, is likely one of the immediate outcomes of supervisor bullying behavior [44]. Building, the following hypothesis can be proposed:

**Hypothesis** **2 (H2).**  *Workplace bullying can positively influence feedback avoidance behavior.*

### 2.3. Feedback Avoidance Behavior (FAB) and Employee Well-Being (EWB)

The conflict between a supervisor and their followers directly indicates avoidance behavior [45], likely adversely impacting employee well-being [40]. Previous evidence has shown that feedback avoidance behavior may act as “a passive coping strategy” for followers to help them manage supervisory abuse (supervisor bullying) [25]. At the same time, passive coping strategy was linked to poor psychological adjustment and depression, as well as unsatisfactory outcomes [46]. Commonly, those who resort to passive coping styles engage in avoidance, withdrawal, and wishful thinking and repeatedly say phrases such as, “It’s awful, and I feel that it overwhelms me,” “I pray to God it won’t last long” [46]. Therefore, feedback avoidance behavior is highly likely to harm employees’ well-being. Accordingly, we can introduce the hypothesis below:

**Hypothesis** **3 (H3).**  *Feedback avoidance behavior can negatively influence employee well-being.*

Drawing on the application of the JD-R model and the conservation of resources (COR) theory, the integration of prior evidence, and the previously outlined rationale for the previous three direct hypotheses among the study’s factors, we can propose the following two hypotheses to address the mediation relationships:

**Hypothesis** **4 (H4).**  *FAB mediates the association between WB and EWB.*

### 2.4. Psychological Safety (PS) as a Moderator

**Figure 1:**
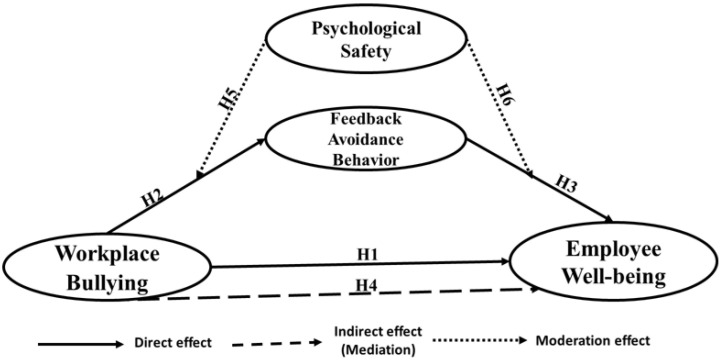
The research model.

Psychological safety was conceptualized by [47] as people’s perception of whether he/she feels comfortable showing and expressing himself/herself without fear of adverse outcomes to career, self-image, or status. He argues that workers are more likely to feel psychologically safe when they encounter supportive and trusting social relationships with their peers in the workplace. Psychological safety was also conceptualized as a shared and rooted belief among people about whether it is safe to participate in interpersonal workplace risk-taking [48,49]. The majority of previous studies widely adopted this conceptualization [50]. Psychological safety was positively linked to enhanced knowledge sharing between team members in the workplace [51,52], higher employee voice behavior [53], and better reporting of treatment mistakes [54] while being negatively associated with silence behaviors [53]. Subsequently, staff with a high sense of psychological safety were more biased in offering honest feedback to their managers [50]. Thus, psychological safety is likely to dampen feedback avoidance behavior. In the same context, the literature on health promotion advocates that psychological safety is an approach to employee well-being. People who perceive high levels of psychological safety are better able to choose the most suitable available coping styles and have heightened levels of well-being [55,56,57]. Thus, the two hypotheses below can be introduced, as pictured in Figure 1.

**Hypothesis** **5 (H5):**  *PS can moderate the impact of WB on FAB.*

**Hypothesis** **6 (H6):**  *PS can moderate the influence of FAB on EWB*

## 3. Methods

### 3.1. Procedures

This study design employed a quantitative research approach to examine the effect of workplace bullying (WB) on employee well-being (EWB), with a focus on the mediating role of feedback avoidance behavior (FAB) and the moderating role of psychological safety (PS). In this study design, a comprehensive review of the relevant literature was conducted to develop the conceptual framework and validated measures from previous studies were used to construct the questionnaire. The finalized questionnaire was then distributed to the target participants. Data were gathered in December 2022 using a convenience sampling technique in conjunction with the drop-and-collect approach. The survey had two stages. Workers responded to WB practices, EWB variables, and demographic details in the first one; then, these employees completed the PS and FAB variables questionnaire after a month, and the collected data were analyzed using PLS-SEM.

### 3.2. Participants

Data were collected via a questionnaire survey targeting Egyptian hotel employees, specifically in Sharm El-Sheikh, due to its numerous high-ranked five-star hotels. Employees with two years of experience or more were allowed to give answers to the study survey as they had enough experience to answer the designed questionnaire adequately. The surveys were distributed with the support of postgraduate researchers from the authors’ institutions employed at the selected hotels. These researchers assisted the authors in reaching out to the hotel HR managers. The HR managers, in turn, communicated the study’s objectives to their staff and distributed the questionnaires for completion after securing approval from the management. The survey was distributed to employees using a social media network with Google Forms link. The questionnaire was voluntary for participants to complete, and their responses were kept confidential. Participants were also informed that agreeing to participate in the survey implied signing the informed consent form. A total of 550 questionnaires were distributed across the two stages. After banning invalid responses, 341 forms were retained, with a response rate of 62%. The sample comprised 276 males (80.9%) and 75 females (19.1%). Most participants (80%) were found to be between 25 and 52 years old.

### 3.3. Measures

The questionnaire consisted of two main sections: the first focused on collecting demographic information about the respondents, while the second included items related to the four constructs outlined in the hypothesized model. All variables’ measures were borrowed from prior research and evaluated on a 5-point Likert scale. The WB was gauged using seven items (α = 0.91) from Einarsen et al. [58]. The sample items include: “Persistent criticism of your work and effort” and “Someone reminding you repeatedly about your errors or mistakes”. The EWB was assessed utilizing six items (α = 0.87) from Zheng et al. [59]. The measure includes items such as: “I am satisfied with my work responsibilities” and “In general, I feel fairly satisfied with my present job”. Five items (α = 0.72) for measuring PS were borrowed from Liang et al. [60]. Sample items include the following: “In my work unit, I can express my true feelings regarding my job”, and “In my work unit, I can freely express my thoughts”. Finally, the six-item scale (α = 0.81) from Moss [39] was used to gauge FAB. The sample items include: “I tried to schedule outside appointments to avoid my supervisor” and “I went the other way when I saw my supervisor coming”. Sixteen academics and practitioners evaluated the questionnaire items in terms of clarity, terminology accuracy, and employees’ ability to understand the measurement items for the study constructs. Based on this evaluation, the content remained unchanged throughout the process.

### 3.4. Analysis of the Study Data

The hypotheses were tested using PLS-SEM with Smart PLS V3.0 software. This study chose PLS-SEM over CB-SEM because it aims to predict one or more variables rather than validate an existing theoretical model, thereby expanding existing structural theory [61]. Additionally, PLS-SEM enables successful testing of more complex models with fewer data constraints and a broader range of sample sizes. The technique is particularly favorable for prioritizing prediction accuracy [62]. Furthermore, PLS is well-suited for both exploratory and confirmatory analyses, as it effectively handles complex and extensive relationships involving multiple constructs and items models [63]. This approach includes two phases: assessing the outer and inner models [64].

## 4. The Results

### 4.1. Measurement Model Results

The outer model was evaluated with regard to convergent validity (CV) and discriminant validity (DV). Cronbach’s alpha, factor loading, and composite reliability should preferably be >0.7, and the Average Variance Extracted (AVE) must be >0.5 to ensure the validity of the outer model [61]. Likewise, regarding DV, the √AVE of the factor should exceed the correlation between that factor and other factors pictured in the model [65], and in addition the Heterotrait–Monotrait ratio of correlation (HTMT) should be <0.9 [66] to confirm the DV of the model. The results in Table 1, Table 2 and Table 3 show that the criteria for achieving CV and DV have been met, thus confirming the outer model’s validity.

### 4.2. Inner Model Assessment

As shown in Table 4, the VIF values were >5, revealing no problem with multicollinearity [61]. Similarly, according to Table 4, our model’s R^2^s were >0.10 [61], and Q^2^s were 0.0 [67]; thus, the proposed model’s explanatory precision was satisfactory.

Some studies [68,69] have used the following equation and the Standardized Root Mean Square Residual (SRMR) in addition to the previous criteria to verify the GoF of the proposed model:

$$\mathrm{GoF}=\sqrt{{AVE}_{avy}\times {R^{2}}_{avy}}$$

It is considered acceptable if the GoF value resulting from the equation exceeds 0.36 [69] and SRMR is <0.08 [70]. After applying the equation to this study’s results, the GoF was found to be 0.622, and the SRMR value was 0.07.

After examining the suitability of the outer and inner models, the beta, t-value, and significance tests were used to test the study hypotheses (Table 5).

**Figure 2:**
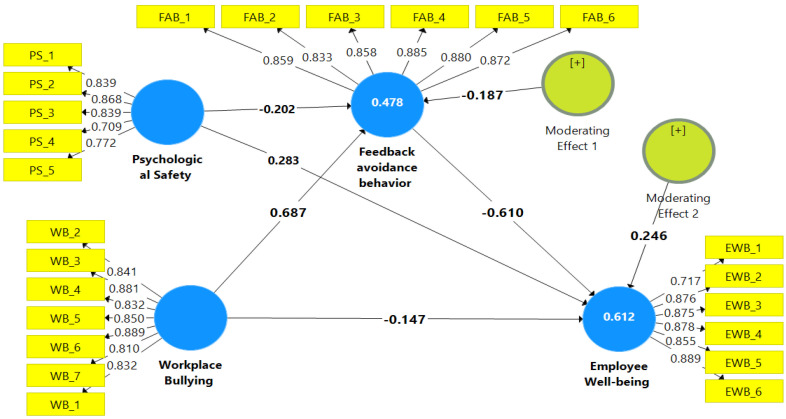
The study model.

Figure 2 illustrates the outer measurement model (for convergent and discriminant validity) and the inner model (for hypothesis testing) as an output from the PLS-SEM v3 program. The outcomes of Figure 2 and Table 5 give evidence that WB negatively affects EWB at β = −0.147, t = 2.607, and *p* < 0.009) and positively affects FAB (β = 0.687, t = 15.136, *p* < 0.000), confirming H1 and H2. The FAB also negatively impacted EWB at β = −0.610, t = 9.238, and *p* < 0.000, guaranteeing H3. Furthermore, FAB successfully mediates the impact of WB on EWB (β = −0.420, t = 10.946, *p* < 0.000), supporting H4.

**Figure 3:**
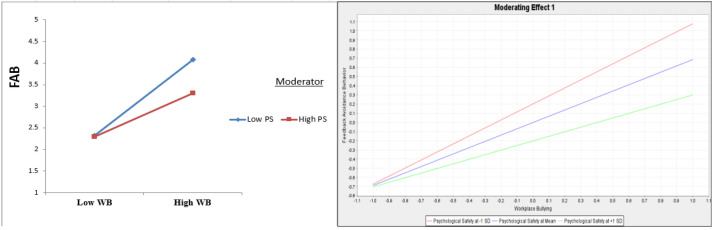
The role of PS as moderator in the link between WB → EWB.

**Figure 4:**
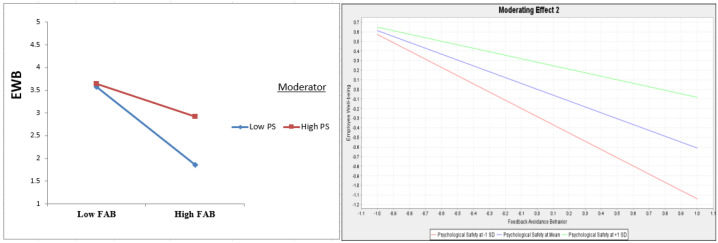
The role of PS as moderator in the link between WP → EWB. Moderator: means the test of the moderation effects.

PS, according to Table 5 and Figure 3 and Figure 4, dampens the impact of WB on FAB (β = −0.187, t = 4.590, and *p* = 0.000) and FAB on EWB (β = 0.246, t = 4.814, and *p* = 0.000), confirming H5 and H6.

## 5. Discussion and Theoretical Implications

Although the harmful outcomes of bullying in the workplace are widely acknowledged, the mechanisms by which these effects are caused by bullying have yet to be sufficiently described in the literature. This restricts our capacity to address the issue of bullying and alleviate its effects on employees who are its targets [71,72]. To respond to this, the current study employed data which were collected from employees in the hotel industry as one of the sectors with the highest documented occurrences of harassment and bullying among all other industries [9] to test the interrelationships of WB, EWB, the FAB as a mediator, and PS as a moderator. For that reason, the outcomes of our paper reached its goals and purposes by contributing to the present literature on workplace bullying and theoretical development via the suggested model. The results demonstrated that WB practices negatively affect EWB (H1). Several studies, according to stress theories, have shown that workplace bullying can result in a drop in job satisfaction, an increase in burnout [73,74,75], lower performance and commitment [76,77], high levels of sickness absenteeism [78,79], and psychotropic drug use [80,81] and, thus, based on the conservation of resources (COR) theory, declining employee well-being rates [82,83,84]. Although the damaging and destructive effect of WB on EWB results in reduced efficiency and service quality, research examining the influences of WB on EWB has paid the tiniest attention to researching the nature of the connection [13]; in addition, little is known about the reasoning mechanisms connecting WB to EWB, more precisely, in the hospitality industry [22]. Thus, our study theoretically contributed to bridging this gap.

Furthermore, employees’ feedback avoidance behaviors may be used as a coping style to avoid stress or to safeguard their already limited resources via avoiding further abuse (i.e., WB) [43]. In line with this, our study proved that the WB positively affected FAB (H2). In fact, prior studies indicated that WB might force employees to use feedback avoidance behaviors (FAB) as a passive coping strategy to handle WB and avoid disciplinary procedures from their leaders [25,43,85]. By operating FAB as a coping style, employees believe they are generating a safe distance, both physically and psychologically, to decrease discomfort with regard to WB [25,86,87]. Generally, workplace feedback behaviors are rarely studied in relation to bullying [88]. On the same statistical path, the study results found that FAB negatively affects EWB (H3). Here, studies asserted that supplying feedback to employees is considered to be crucial for maintaining and improving employee motivation and satisfaction, and thus, EWB, and vice versa [89,90]. Therefore, depending on social exchange theory (SET), supervisors might adopt several specific behaviors to reinforce organizational feedback operations, improve leader/subordinate relationships, and boost employee satisfaction and well-being [90].

The current study sought to test whether FAB mediates the association between WB and EWB (H4). The study’s findings, which confirmed the three hypotheses mentioned above, revealed that FAB was indeed a mediator between WB and EWB. Based on previous studies, we argue that employees’ use of FAB as a passive coping style due to WB behaviors [43] will lead to destructive and catastrophic effects on EWB [90].

Finally, our PLS-SEM results confirmed the moderation impact of PS on the links between WB and FAB (H5) and also on the links between FAB and EWB (H6). In the health promotion literature, scholars generally endorse psychological safety (PS) as an approach to support EWB [56,57]. Employees who perceive heightened levels of PS can better determine the most suitable available coping strategy to reach a high level of EWB [55,91]. Therefore, a PS climate is predicted to promote increased levels of EWB compared to an unsafe work environment since PS makes employees feel comfortable and unrestricted from external controls or restrictions and engages them in more positive behavior [92,93]. Accordingly, the PS variable succeeded in alleviating (moderating) the positive relationship of the WB toward FAB and the negative linkage of the FAB toward EWB. Managers must confront WB behaviors to reduce FABs to improve EWB. Improving the PS climate can be used in this task to alleviate the negative consequences.

## 6. Theoretical Implications

Based on the outcomes of the current study, which revealed the direct negative impact of WB on EWB, as well as its role in promoting employee feedback avoidance among employees due to fears of resource loss resulting from bullying behaviors, it is evident that feedback avoidance serves as a mediating factor exacerbated by workplace bullying, and, in turn, contributes to the deterioration of employee well-being. In addition, the study used psychological safety as a moderator that could contribute to mitigating the positive impact of WB on feedback avoidance behavior as well as the negative impact of WB on employee well-being. The study recommends designing workshops and training sessions to raise employees’ and managers’ awareness of WB, its consequences, and strategies for effectively recognizing and addressing such behaviors. It is also essential to provide a work environment that supports psychological safety, through which employees can be encouraged to express their opinions and concerns without fear of retaliation. This can be achieved by training managers and supervisors to actively listen to their employees and provide guidance and emotional support when needed. Regular training sessions highlighting the importance of psychological safety, including determining bullying behaviors, handling them effectively, and promoting a culture of respect and inclusion, are also essential. Furthermore, to improve the climate of psychological safety, transparent and clear systems and policies for requesting feedback and reducing the avoidance of it due to fear of bullying behaviors from supervisors must be established. Here, it is recommended that a continuous employee feedback system be designed that allows employees to share their concerns and experiences in a confidential and safe manner. This will help identify potential issues before they escalate and strengthen employees’ sense of security.

The interrelationships investigated between workplace bullying, staff well-being, feedback avoidance, and psychological safety are not exclusive to the hotel industry. These challenges have existed in various sectors and industries (i.e., education, healthcare, finance, retail, and manufacturing operations). Investigating such connections in other contexts (industries and countries) might highlight the universality of the investigated issue and allocating the research finding as more widely applicable.

## 7. Conclusions

This paper strived to explore the interrelationships between workplace bullying (WB), employee well-being (EWB), feedback avoidance behavior (FAB), and psychological safety (PS). By using PLS-SEM, the data collected from hotel employees were analyzed. The study results displayed that WB negatively affects EWB and positively affects FAB, while FAB has a negative impact on EWB. Moreover, FAB successfully mediated the impact of WB on EWB, and PS also successfully moderated the effect of WB on FAB and FAB on EWB. Based on these findings, the study has made a theoretical contribution by utilizing the Job Demands–Resources (JD-R) model in the literature on hospitality industry organizational climate. Additionally, the study offers practical applications to assist hotel managers in addressing workplace bullying and its adverse impact on employee well-being, including their tendency to avoid feedback due to such bullying. It also highlights how psychological safety can be leveraged to improve the work environment by mitigating the adverse effects of workplace bullying.

## 8. Limitations

While this paper has valuable implications for the dynamics of information about workplace bullying in the tourism hospitality industry, several limitations can be recognized. First, this study’s main limitation was the use of a convenience sampling method, which may not fully capture a diverse cross-section of hotel employees. Future research should consider using a probability sampling method to examine the correlations between the variables explored in this study. Also, the cross-sectional study design limits the capacity to infer causal relationships between the study variables. Future research papers can implement a longitudinal research design better to understand the causal interrelationship between the research variables. Second, the collected data were from hotel employees in Egypt, which can limit the capability to generalize the revealed findings to other contexts. Considering the universal nature of the tourism and hospitality industry, future research papers may investigate information flow about workplace bullying and its expected outcomes across diverse countries and context to understand these interrelationships better. Finally, this research paper mainly investigated employees’ perceptions of workplace bullying, which may be induced by personal biases or subjective judgements of workplace practices. Integrating other sources of data, such as managers’ or supervisors’ perceptions and perspectives, can provide a more holistic and comprehensive overview of workplace bullying and its influence on employee well-being.

## Figures and Tables

**Table 1:** Scale validity and reliability.

	Loadings	*a* Value	C.R	AVE
Workplace Bullying (WB)		0.935	0.947	0.720
WB1	0.832			
WB2	0.841			
WB3	0.881			
WB4	0.832			
WB5	0.850			
WB6	0.889			
WB7	0.810			
Psychological Safety (PS)		0.869	0.903	0.652
PS1	0.839			
PS2	0.868			
PS3	0.839			
PS4	0.709			
PS5	0.772			
Feedback Avoidance Behavior (FAB)		0.932	0.947	0.748
FAB_1	0.859			
FAB_2	0.833			
FAB_3	0.858			
FAB_4	0.885			
FAB_5	0.880			
FAB_6	0.872			
Employee Well-being (EWB)		0.923	0.940	0.723
EWB1	0.717			
EWB2	0.876			
EWB3	0.875			
EWB4	0.878			
EWB5	0.855			
EWB6	0.889			

**Table 4:** VIF, R^2^, and Q^2^ results.

Indicator	VIF	Indicator	VIF	Indicator	VIF	Indicator	VIF
WB1	3.269	PS1	2.104	FAB1	2.778	EWB1	1.846
WB2	3.532	PS2	2.366	FAB2	2.499	EWB2	4.231
WB3	3.371	PS3	2.061	FAB3	2.940	EWB3	4.016
WB4	3.110	PS4	1.812	FAB4	3.198	EWB4	3.050
WB5	3.362	PS5	2.076	FAB5	3.115	EWB5	2.634
WB6	4.355			FAB6	3.023	EWB6	3.290
WB7	2.614						
Employee Well-being (EWB)				R^2^	0.612	Q^2^	0.427
Feedback avoidance behavior (FAB)				R^2^	0.478	Q^2^	0.350

**Table 5:** Hypothesis analysis.

Hypotheses	β	t-Value	*p*-Values	Findings
Direct Paths				
H1: WB → EWB	−0.147	2.607	0.009	Confirmed
H2: WB → FAB	0.687	15.136	0.000	Confirmed
H3: FAB → EWB	−0.610	9.238	0.000	Confirmed
Indirect mediating Paths				
H4: WB → FAB → EWB	−0.420	10.946	0.000	Confirmed
Moderating Effects				
H5: WB × PS → FAB	−0.187	4.590	0.000	Confirmed
H6: FAV × PS → EWB	0.246	4.814	0.000	Confirmed

**Table 2:** Fornell–Larcker matrix.

	EWB	FAB	PS	WB
Employee Well-being (EWB)	**0.850**			
Feedback avoidance behavior (FAB)	−0.657	**0.865**		
Psychological Safety (PS)	0.459	−0.311	**0.807**	
Workplace Bullying (WB)	−0.617	0.649	−0.405	**0.848**

Note: Bold values are the √AVE.

**Table 3:** HTMT Matrix.

	EWB	FAB	PS	WB
Employee Well-being (EWB)				
Feedback avoidance behavior (FAB)	0.693			
Psychological Safety (PS)	0.485	0.328		
Workplace Bullying (WB)	0.641	0.683	0.429	

Note: all HTMTs < 0.90.

## Data Availability

The data presented in this study are available on request from the corresponding author.
